# Evaluation of Bachmann Bundle Pacing Versus Right Atrial Pacing in Prevention of Atrial Fibrillation After Coronary Artery Bypass Surgery

**Published:** 2011-02-07

**Authors:** Chandrakant Chavan, Mukund Karmalkar, Rajesh Badani, K Sharada, Usha Rani, Prasad Rao, Ram Subramanyam, C Narasimhan

**Affiliations:** CARE Hospitals, The Institute of Medical Sciences, Hyderabad

**Keywords:** atrial fibrillation, pacing, Bachmann bundle

## Abstract

**Background:**

In patients undergoing coronary artery bypass surgery (CABGS), occurrence of atrial fibrillation (AF) is common in the postoperative period and is associated with increased morbidity with longer intensive unit care (ICU) and hospital stay. Prevention with antiarrhythmic drugs is of limited success and associated with significant side effects. Therefore alternative approaches, such as Bachmann Bundle pacing, are required.

**Methods and Results:**

154 consecutive patients, mean age 58±8.8 years, including 134 males and 20 females, were randomized to three groups; Group I : No pacing n= 54, Group II : RA pacing n= 52, Group III : Bachmann Bundle pacing  n= 48. All the groups were well matched with regard to age, left atrial size, ejection fraction and use of beta blockers. Patients in Groups II and III were continually paced at a rate of 100 beats per minute (bpm) or at 10 bpm more than patients' intrinsic heart rate. All the patients were monitored for 72 hours by telemetry and occurrence of AF was noted. Incidence of AF was 0% (none of 48 patients) in Group III as compared to 16.6% in Group I (9 of 54 patients) (p 0.003) and 12.5% in Group II (5 of 52 patients) (p 0.03). There was a trend towards shorter ICU stay in Group III (3.9 days) as compared to Group II (4.5 days) and Group I (4.1 days). Among the three groups, the reduction in mean P wave duration also was greater in Bachmann bundle paced group.

**Conclusion:**

In patients undergoing CABGS, Bachmann bundle pacing is superior to right atrial / no pacing in the post operative period for preventing occurrence of AF and reducing ICU stay, commensurate with a reduction in mean P wave duration on surface ECG.

## Introduction

Postoperative atrial fibrillation (AF) is a common complication of cardiac surgery, occurring in 25% to 40% of patients [1]. Several reports have indicated that the postoperative AF has been associated with increased hospital length of stay (LOS), increased rates of postoperative stroke, and, in consequence, an increased total cost of surgery [1,2]. Chronic AF is convincingly shown to cause stroke, and, consequently, prevention of postoperative AF could reduce the risk of this major morbid outcome. Prevention of AF is a reasonable clinical goal, and, consequently, many randomized trials [17,18,25,26]  have evaluated the effectiveness of pharmacological and non-pharmacological interventions for prevention of AF. This randomized controlled study was conducted with the hypothesis that Bachmann bundle (BB) site pacing is superior to right atrial (RA) pacing as the atrial activation is more homogenous due to simultaneous and synchronized activation of both the atria.

## Patients and Methods

Consecutive patients undergoing coronary artery bypass surgery with sinus rhythm were included in the study.   Patient selection criteria were, need of CABG for angina relief or recurrent heart failure with triple vessel disease, LAD disease was mandatory for randomization, age > 40 years, LVEF > 35%, left atrial size less than 55 mm in parasternal long axis view on echocardiography. Patients with chronic atrial fibrillation, persistent sinus tachycardia (> 100 beats/min.), pre-operative pacemaker dependency, amiodarone therapy for more than two weeks in the past one-year, congenital heart disease, cardiac neoplasm were excluded from study. Patients were randomized to three groups; Group I was control group (54 patients): no pacing was done postoperatively in these patients. Group II (52 patients): right atrial pacing was done using epicardial wire on right atrial appendage. These patients were continuously paced at 100 bpm or 10 bpm above the intrinsic heart rate, whichever was higher. Group III: (Study group- 48 patients): continuous pacing of Bachmann's bundle was done using the same protocol as in Group II through epicardial approach. Bachman bundle was paced through wire placed epicardially in the superior portion of interatrial septum (IAS). This site was accessed after lifting up the right atrial appendage and palpating posterior and left portion of aorta and wire was placed at tough part of IAS. Pacing was done for 72 hours in-group II and III patients. The study was approved by the institutional review board. All patients were monitored with telemetry for occurrence of AF. Also we studied the influence of pacing on P wave duration in each patient. Three patients's had dislodgment of pacing wire (increase in pacing threshold more than two times at the time of implant). Overall 7 patients had increase in threshold; in four patients it was not significant. There were no complications related to epicardial wire removal.

## Results

154 consecutive patients undergoing coronary artery bypass surgery with sinus rhythm were included in the study. Of these, 54, 52, and 48 were assigned to the no pacing, right atrial pacing and Bachmann Bundle pacing groups, respectively. The mean age was 58#8.8 years, and 87 % of the patients were male. All the groups were well matched with regard to age, left atrial size, ejection fraction and use of beta blockers (Table 1). Majority (85%) of patients were taking beta blockers before the surgery and this was not significantly different among the 3 groups.

### Effect of Atrial Pacing on P-Wave Analysis

**Figure 1:**
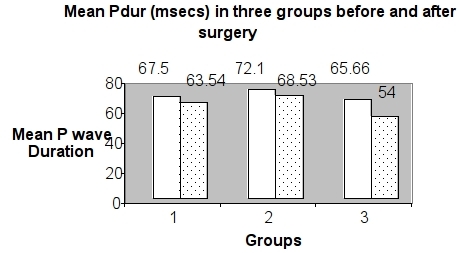
Mean P wave duration (Pdur) in three different groups before (clear bar) and after operation(dotted bar).

The relationship between delayed atrial conduction times and P wave duration (Pdur) has led to an analysis of standard or signal-averaged ECG Pdur as a prognostic index for the development of AF after cardiac surgery [3-7].   We sought to determine if mean Pdur  from a standard ECG could provide a simple yet effective measurement of delayed and nonuniform atrial conduction in post-operative AF. At baseline, no significant differences existed in the mean Pdur (Group I 67.5 ± 23.14, Group II 72.19 ± 21.27, Group III 65.66 ± 14.30). The mean Pdur was significantly reduced in Bachmann's bundle pacing group after surgery (Figure 1).

### Incidence of Postoperative AF

**Figure 2:**
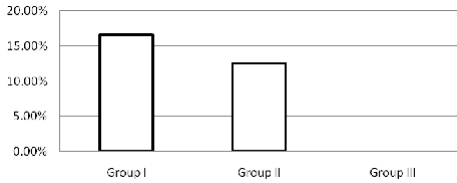
Incidence of atrial fibrillation in three different groups in post coronary artrery bypass surgery, Group I: No pacing n= 54 (16.6%), Group II: RA pacing n= 52 (12.5%), Group III: Bachmann Bundle pacing n= 48 (0%).

A total of 14 patients developed AF, with an overall incidence of 9.09%. There was a significantly reduced incidence of postoperative AF in the Group III (0%) when compared with the other two groups: RA pacing 12.5% (p 0.003) and No pacing 16.6%  (p 0.03). If AF was not converted spontaneously to sinus rhythm (SR), either pharmacological means or electrical cardioversion was used to restore SR before discharge. After 4 weeks of follow-up, all patients remained in SR. The mean length of stay in the intensive care unit was also significantly reduced in Group III (3.9 days) as compared to Group II (4.5 days) and Group I (4.1 days). (Figure 2)

## Discussion

Previous studies demonstrated that use of beta-blockers and sotalol have beneficial effect in prevention of postoperative AF [8,9]. Daoud and associates showed that preoperative amiodarone therapy was effective in reduction of postoperative AF [10]. Medical therapy as a prophylactic agent against post-CABG AF, may be limited by other medical disease, such as asthma, thyroid dsyfunction, or liver function derangement [11,12].

We demonstrated that Bachmann bundle overdrive pacing is more effective in reducing the incidence of AF after CABG than control and single-site right atrial pacing. This type of pacing was also associated with a significantly reduced length of hospitalization. Atrial overdrive pacing from Bachmann bundle site reduced the mean Pdur significantly. Patients who develop AF have a prolonged postoperative hospital stay compared with patients who remain in sinus rhythm; this finding was confirmed in the present study. Recent evidence shows that prolonged hospitalization is attributable to arrhythmia rather than clinical characteristics of AF patients [8,13,14,15]. Overdrive atrial pacing has been proposed for the prevention of AF recurrence in patients with bradycardia and paroxysmal AF [16]. Because temporary pacing leads are routinely used after open-heart surgery, there have been some preliminary reports on the effectiveness of atrial pacing in preventing post-CABG AF [17,18]. Biatrial pacing was introduced as a new pacing modality for the prevention of atrial tachyarrhythmias in patients with AF and advanced interatrial conduction block [19], but the actual incidence of advanced interatrial conduction block is rare in the general population, with a prevalence estimated at 1% [20].

Animal studies demonstrated that dispersion of refractoriness and anisotropic conduction are two essential elements for sustaining atrial arrhythmia [21,22], and both have been implicated in the pathogenesis of postoperative AF [23]. Pacing at a higher rate can suppress bradycardia-induced atrial premature contractions and may reduce the dispersion of refractory periods [24]. Unique pacing sites can "pre-excite" the abnormal substrate and, subsequently, increase the coupling interval of activation by a premature beat, thus preventing the initiation of reentry. Au et al.  performed a small case-control study that showed no difference between biatrial pacing and controls in two groups of 52 patients. This study was however very small and non-randomized [25]. A randomized trial with biatrial pacing or controls showed the incidence of AF to be 17.9% in the biatrial pacing group compared to an incidence of 33.9% in the control group [26]. Bachmann bundle pacing results in homogeneous activation of both atria and maintains intraatrial and interatrial synchrony [8,11,27]. This may help to prevent atrial fibrillation in this subgroup of patients [30].

The economic consequences of post-CABG complications were studied [14,28,29].  It showed that AF was one of the less expensive but most common complications, occurring in 20% of patients [29]. Bachmann Bundle pacing reduces hospital stay, reducing the morbidity related to AF and economic burden.

## Conclusion

AF is commonly encountered after CABGS, and it results in an increased hospital stay. In patients undergoing coronary artery bypass surgery, Bachmann bundle pacing results in a significant reduction in mean P wave duration on surface ECG and is superior to right atrial / no pacing in the post operative period for preventing occurrence of atrial fibrillation and reducing intensive care unit stay.

## Figures and Tables

**Table 1:**
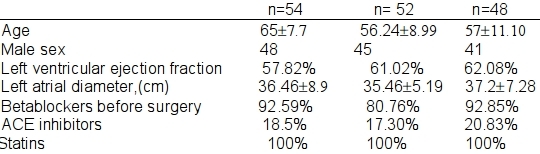
Clinical Characteristics of the Patients

## References

[R1] Ommen SR (1997). Atrial arrhythmias after cardiothoracic surgery. N Engl J Med.

[R2] Roach GW (1996). Adverse cerebral outcomes after coronary bypass surgery. N Engl J Med.

[R3] Stafford PJ (1997). Signal averaged P wave compared with standard electrocardiography or echocardiography for prediction of atrial fibrillation after coronary bypass grafting. Heart.

[R4] Steinberg JS (1993). The value of P-wave signal averaged  electrocardiogram for predicting atrial fibrillation after cardiac surgery. Circulation.

[R5] Frost L (1996). Re-evaluation of the role of P-wave  duration and morphology as predictors of atrial fibrillation and flutter after coronary artery bypass surgery. Eur Heart J.

[R6] Seifert M (1993). P-wave signal averaging: high tech or an expensive alternative to standard ECG?. Circulation.

[R7] Cresewell LL (1993). Hazards of postoperative atrial arrhythmias. Ann Thorac Surg.

[R8] Wolman RL (1999). Cerebral injury after cardiac surgery: indication of a group at extraordinary risk. Stroke.

[R9] Cardona F (2003). Effect of right atrial pacing, intravenous amiodarone and beta blockers for suppression of atrial fibrillation after coronary artery bypass surgery: a pilot study. P R Health Sci J.

[R10] Daoud EG (1997). Preoperative amiodarone as prophlaxis against atrial fibrillation after heart surgery. N Engl J Med.

[R11] Katherine Fan (2000). Effects of Biatrial Pacing in  Prevention of postoperative Atrial Fibrillation After Coronary Artery Bypass Surgery. Circulation.

[R12] White CM (2003). Atrial Fibrillation Suppression Trial II. Intravenous plus oral amiodarone, atrial septal pacing, or both strategies to prevent post-cardiothoracic surgery atrial fibrillation: the Atrial Fibrillation Suppression Trial II (AFIST II). Circulation.

[R13] Taylor GJ (1987). Usefulness of atrial fibrillation as a predictor of stroke after isolated coronary artery bypass grafting. Am J  Cardiol.

[R14] Aranki SF (1996). Predictors of atrial fibrillation after coronary artery surgery: current trends and impact on hospital resources. Circulation.

[R15] Kubara I (1999). Dispersion of filtered P wave duration by P wave signal-averaged ECG mapping system: its usefulness for determining efficacy of disopyramide on paroxysmal atrial fibrillation. J Cardiovasc Electrophysiol.

[R16] Garrigue S (1998). Prevention of atrial arrhythmias during DDD pacing by atrial overdrive. Pacing Clin Electrophysiol.

[R17] Gerstenfeld EP (1999). Evaluation of right atrial and biatrial temporary pacing for the prevention of atrial fibrillation after coronary artery bypass surgery. J Am Coll Cardiol.

[R18] Kurz DJ (1999). Epicardial biatrial synchronous pacing for prevention of atrial fibrillation after cardiac surgery. Pacing Clin Electrophysiol.

[R19] Daubert C (1994). Atrial tachyarrhythmias associated with high degree interatrial conduction block: prevention by permanent atrial resynchronization. Eur J Card Pacing Electrophysiol.

[R20] Bayes de Luna A (1988). Interatrial conduction block  and retrograde activation of the left atrium and paroxysmal supraventricular tachyarrhythmia. Eur Heart J.

[R21] Spach MS (1988). Influence of the passive anisotropic properties on directional differences in propagation following modification of  the sodium conductance on human atrial muscle: a model of reentry based on anisotropic discontinuous propagation. Circ Res.

[R22] Allessie MA (1976). Circus movement in rabbit atrial muscle as a mechanism of tachycardia, II: the role of nonuniform recovery of excitability in the occurrence of unidirectional block, as studied with multiple microelectrodes. Circ Res.

[R23] Sato S (1992). The effect of augmented atrial hypothermia on atrial refractory period, conduction, and atrial flutter/ fibrillation in the canine heart. J Thorac Cardiovasc Surg.

[R24] Han J (1966). Temporal dispersion of recovery of excitability in atrium and ventricle as a function of heart rate. Am Heart J.

[R25] Au WK (2003). Biatrial Pacing to prevent Atrial Fibrillation after Coronary Artery Bypass. Asian Cardiovascular and Thoracic Annals.

[R26] Orr W (V). Synchronised biatrial pacing after coronary artery bypass surgery. Pacing and Clinical Electrophysiology.

[R27] Archbold RA (2004). Atrial Pacing for the prevention of atrial fibrillation after coronary artery bypass graft surgery: A review of the literature. Heart.

[R28] Mauldin PD (1994). Predicting hospital costs for first time coronary artery bypass grafting from preoperative and postoperative variables. Am J Cardiol.

[R29] Taylor GJ (1990). Determinants of hospital charges for coronary artery bypass surgery: the economic consequences of postoperative complications. Am J Cardiol.

[R30] Goette Andreas (2002). Pacing of Bachmann's Bundle After Coronary Artery Bypass Grafting. Pacing and Clinical Electrophysiology.

